# Assessment of Exercise Intensity for Uphill Walking in Healthy Adults Performed Indoors and Outdoors

**DOI:** 10.3390/ijerph192416662

**Published:** 2022-12-12

**Authors:** Laura Eisenberger, Barbara Mayr, Maximilian Beck, Verena Venek, Christina Kranzinger, Andrea Menzl, Inga Jahn, Mahdi Sareban, Renate Oberhoffer-Fritz, Josef Niebauer, Birgit Böhm

**Affiliations:** 1 Institute of Preventive Pediatrics, Faculty of Sport and Health Sciences, Technical University of Munich, 80992 Munich, Germany; 2 Institute of Sports Medicine, Prevention and Rehabilitation and Research Institute of Molecular Sports Medicine and Rehabilitation, Paracelsus Medical University, 5020 Salzburg, Austria; 3 Ludwig Boltzmann Institute for Digital Health and Prevention, 5020 Salzburg, Austria; 4 Salzburg Research Forschungsgesellschaft mbH, 5020 Salzburg, Austria; 5 Department of Medical Engineering, Carinthia University of Applied Sciences, 9524 Villach, Austria; 6 St. Irmingard Klinik Prien, Clinic for Cardiology, 83209 Prien am Chiemsee, Germany

**Keywords:** self-perception, modified Bruce protocol, cardiorespiratory fitness, exercise testing, prevention, hiking, natural environment, physical activity

## Abstract

Background: Borg’s rating of perceived exertion (BRPE) scale is a simple, but subjective tool to grade physical strain during exercise. As a result, it is widely used for the prescription of exercise intensity, especially for cardiovascular disease prevention. The purpose of this study was to assess and compare relationships between BRPE and physiological measures of exercise intensity during uphill walking indoors and outdoors. Methods: 134 healthy participants [median age: 56 years (IQR 52–63)] completed a maximal graded walking test indoors on a treadmill using the modified Bruce protocol, and a submaximal 1 km outdoor uphill cardio-trekking test (1 km CTT). Heart rate (HR) and oxygen consumption (V̇O_2_) were continuously measured throughout both tests. BRPE was simultaneously assessed at the end of each increment on the treadmill, while the maximal BRPE value was noted at the end of the 1 km CTT. Results: On the treadmill, BRPE correlated very high with relative HR (%HR_max_) (ρ = 0.88, *p* < 0.001) and V̇O_2_ (%V̇O_2max_) (ρ = 0.89, *p* < 0.001). During the 1 km CTT, a small correlation between BRPE and %HR_max_ (ρ = 0.24, *p* < 0.05), respectively %V̇O_2max_ was found (ρ = 0.24, *p* < 0.05). Conclusions: Criterion validity of BRPE during uphill walking depends on the environment and is higher during a treadmill test compared to a natural environment. Adding sensor-based, objective exercise-intensity parameters such as HR holds promise to improve intensity prescription and health safety during uphill walking in a natural environment.

## 1. Introduction

Cardiovascular diseases (CVD) are a significant health problem worldwide, despite the fact that several risk factors are modifiable [1,2]. The prevalence of CVD almost doubled between 1990 and 2019, from 271 to 523 million cases [2]. A healthy lifestyle that includes regular physical exercise is an important preventive strategy [3,4]. Physical activity (PA) clearly has numerous benefits for preventing CVD, as reported in several sets of guidelines [5,6,7]. Unfortunately, in modern societies, the general population spends too little time on physical activity (PA), and therefore, there is an urgent need to improve aerobic exercise in order to reduce CVD burden [8,9,10].

To assess the PA intensity during exercise interventions, tools are required. Cardiopulmonary exercise testing (CPET) is the criterion measurement to assess exercise capacity objectively, and is an essential and widely used tool in clinical applications to prescribe exercise intensity, especially for patients with CVD [11]. Exercise tests have been around for more than 50 years [12]. Despite its methodological complexity and high cost, ventilatory gas analysis is a very popular and accurate test method for the objective assessment of exercise intensity [13]. Instead, subjectively experienced intensity of effort while being physically active such as the Borg’s Rating of Perceived Exertion (BRPE) [14,15,16] is commonly used to assess and prescribe exercise intensity.

The rationale for Borg’s 6–20 scale was based on the linear increase of the values of heart rate (HR) and oxygen consumption (V̇O_2_) during cycling and running [17]. Only a few studies investigated the relationship during walking, although walking is the most often reported PA among adults [18]. Previous findings demonstrated the usefulness of Borg’s 6–20 scale as a tool for prescribing and monitoring exercise intensity from young to elderly participants [19,20,21,22,23]. Additionally, several studies have shown a clear benefit of using BRPE to control exercise intensity for both men and women [24,25,26]. According to the National Institutes of Health Development Panel on Physical Activity and Cardiovascular Health (USA), many people do not see walking as appropriate moderate-level exercise with preventive effects [27]. Although there is substantial evidence that moderate PA, such as brisk walking, is sufficient to reduce the risk of CVD in all male and female age groups, as well as in healthy and patient populations [28,29,30,31], there is still a lack of appreciation for the benefits related to walking. However, walking is reported to be the most common PA (15.0% to 41.8%) among adults in most countries [18] and can help reduce physical inactivity [29]. Notably, hiking which usually consists of walking uphill is gaining popularity, even among elderly individuals [32,33], and is considered to be beneficial to health [34,35]. Thus, exercise testing protocols using walking/hiking yield advantages compared to running.

There have been studies using BRPE in the laboratory to validate exercise intensity assessment; nevertheless, no study has been performed within a more ecologically valid environment, such as outdoor hiking trails. The use of BRPE in a clinical setting supports patients to monitor their exercise intensity if maximal testing is not possible [36]. BRPE has been shown to be a good predictor of exercise intensity in young healthy adults [20,21,24], middle-aged [37,38] and elderly adults [25,39]. In addition, the control of exercise intensity by using BRPE in young and healthy adults leads to cardiorespiratory and muscular fitness improvements [40]. Therefore, exercise recommendations for a patient, as well as a healthy population, already include BRPE [36,41,42,43]. To this end, we set out to examine the relationship between the BRPE scale and physiological measures of exercise intensity during a graded walking test on the treadmill and a 1 km cardio-trekking test (1 km CTT) on an uphill terrain in nature.

## 2. Materials and Methods

### 2.1. Participants and Study Design

We included 134 healthy adults [median age: 56 years (IQR 52–63)], 58 men [median age: 56 years (IQR 52–62)], and 76 women [median age: 57 years (IQR 51–63)] in the Connect2Move project [44,45]. The European cross-border study aimed to highlight natural and safe cardio-trekking trails for the sustainable promotion of cross-generational and health-oriented tourism. Study participants aged ≥45 years were screened by detailed physical and cardiac examinations. These examinations included an anamnesis, anthropometric and blood pressure measurements, a fastening blood sample, pulmonary function testing, resting electrocardiography, and an echocardiography in order to exclude those with cardiovascular or chronic diseases. The Technical University of Munich (Germany) and the Ludwig Boltzmann Institute for Digital Health and Prevention in Salzburg (Austria) conducted the laboratory and field testing. A detailed description of the study design, the inclusion criteria, and the recruitment process is provided elsewhere [44].

The study was performed in accordance with the Declaration of Helsinki and its latest amendments and was approved by the Ethical Committee of the State of Salzburg (EK-Nr.:1090/2020) and the Ethics Committee of the Faculty of Medicine of the Technical University of Munich (527/20S). The present project was registered at ClinicalTrials.gov (NCT05226806). All participants provided written informed consent before their study participation.

### 2.2. Walking Test Settings

After medical examinations, all participants performed a stepwise incremental walking test on the treadmill (h/p/cosmos Sports & Medical GmbH, Nussdorf-Traunstein, Germany) using the modified Bruce protocol [46] (Table 1) until physical exertion. The Bruce protocol is a treadmill test, where both speed and incline is increased every three minutes. The modified Bruce protocol includes two additional stages as a warm-up (stage one and two with 2.7 km·h^−1^ and 0% incline, respectively 5% incline) in addition to the original Bruce protocol. The participants were instructed to walk as long and fast as possible without running. The treadmill was stopped if participants reached maximal exhaustion or started running. BRPE was recorded at each stage. Maximal exhaustion was assumed when the participants achieved at least two of the following four criteria [47,48]:

(1) Maximal Borg value of at least 18 (Borg_max_ ≥ 18);
(2) Respiratory exchange ratio of at least 1.1 (RER ≥ 1.1);
(3) Maximal heart rate of at least 85% of the age-predicted HR_max_ using the equation: 220–age (HR_max_ ≥ 85%);
(4) Leveling-off oxygen consumption despite an increasing workload, increase in O_2_ ≤ 150 mL·min^−1^.

A day after the laboratory testing, all participants performed a submaximal 1 km CTT outdoors either in Aschau im Chiemgau (Germany) or in Werfenweng (Austria) controlled by the 6–20 BRPE scale (Table 2). The 1 km CTT was an uphill outdoor walking test at moderate altitude (<3000 m) [49] with a maximum incline of 26% [45]. Participants were instructed to reach a submaximal effort with a maximum value of 15 (“hard”) on the Borg’s scale throughout the entire 1 km CTT. At the end of the 1 km CTT, the participants were asked to rate their maximal BRPE (BRPE_max_) during the test. The CTT was postponed in adverse weather conditions, e.g., at temperatures above 26 degrees, to avoid the influence of environmental-related risk factors [44,50,51].

### 2.3. Exercise Intensity Measurements

During indoor and outdoor testing HR was measured using a Garmin HRM-Dual chest strap (Garmin, Olathe, KS, USA), and V̇O_2_ was measured using a portable spirometry device (K5, COSMED Deutschland GmbH, Fridolfing, Germany) in dynamic mixing chamber mode (DMC) [53] to objectively determine exercise intensity. BRPE was assessed to determine the subjective exercise intensity of the participants during the two test settings (indoors and outdoors). Before starting the walking test on the treadmill, BRPE 6–20 scale [54] was explained in detail to each subject by the study staff. Participants were asked to assess their BRPE on the scale at the end of every stage of the modified Bruce protocol (every third minute) and the end of the exercise test. In the 1 km CTT, the exercise intensity was rated by the BRPE scale, with a target value of 15/20. The study team continuously showed the scale to the participants to ensure submaximal intensity during the 1 km CTT. At the end of the test, the maximal BRPE was documented.

### 2.4. Statistical Analysis

The statistical analyses were performed using the software IBM SPSS Statistics version 28 (SPSS, Inc., Chicago, IL, USA), and *p* values < 0.05 (two-sided) were considered statistically significant. Data were corrected by identifying errors or outliers. For further statistical analysis, due to the DMC mode, V̇O_2_ values were taken 30 s after each stage change. HR and Borg values were noted at the end of each stage. All variables were tested for normal distribution by using the Kolmogorov–Smirnov test. For the descriptive analyses, means and standard deviations [SD] for normal distribution and medians and interquartile ranges [IQR, 25th–75th percentiles] for non-normal distribution were reported. Data are expressed as the mean ± SD or median + IQR. The statistical significance of the means of the descriptive results of men and women was tested by Student’s *t*-tests for normally distributed data or Mann–Whitney U tests for non-normally distributed data. Linear regression analysis models were performed for all BRPE values and the corresponding HRs and V̇O_2_ values in the laboratory. It was confirmed that the assumptions of linear regression models held. The adjusted coefficient of determination (adjusted r^2^) was used to illustrate the goodness of fit. Correlations were calculated with Spearman’s correlation (Spearman’s rho, ρ) for the treadmill walking testing and the 1 km CTT if at least one variable was non-parametric. The cut-off points for the interpretation of the strength of the correlations were reported by Hopkins [55].

## 3. Results

Characteristics of the participants are presented in Table 3. In total, 27.6% (*n* = 37, men = 25, women = 12), and 3.7% (*n* = 5, men = 3, women = 2) of them had a body mass index (BMI) of 25–30 kg/m^2^, and 30–35 kg/m^2^, respectively. On average, women had a significantly lower BMI than men, *p* < 0.001.

### 3.1. HR, V̇O_2_, and BRPE Measurements during the Walking Tests

All participants fulfilled at least two out of our four criteria of maximal exhaustion. The performance parameters are presented in Table 4. The total cohort (TC) achieved a mean relative V̇O_2max_ of 39.3 ± 7.7 mL·min^−1^·kg^−1^ during treadmill walking testing and a mean relative V̇O_2peak_ of 37.3 ± 6.3 mL·min^−1^·kg^−1^ in the submaximal 1 km CTT. Male participants [median age: 56 years (IQR 52–62)] showed a mean relative V̇O_2max_ of 42.8 ± 8.6 mL·min^−1^·kg^−1^ in the treadmill walking test and female participants [median age: 57 (IQR 51–63)] showed a mean relative V̇O_2max_ of 36.6 ± 5.8 mL·min^−1^·kg^−1^. During both the treadmill and 1 km CTT testing, women had significantly lower cardiorespiratory fitness than men (*p* < 0.001).

Women showed significantly higher HR_peak_ during the submaximal 1 km CTT than men (*p* < 0.01). During both test settings, male participants had significantly higher maximal walking speeds than women (*p* < 0.001 on the treadmill, and *p* < 0.05 outdoors).

At the end of the walking test on the treadmill, seven participants (5.2%) reported a BRPE_max_ value of 20/20 on the Borg 6–20 scale. Most of the participants (*n* = 98, 73.1%) reported a BRPE_max_ value between 17/20 and 19/20. Twenty-nine participants (21.6%) reported a BRPE_max_ value between 14/20 and 16/20.

At the end of the submaximal 1 km CTT, 98 participants (71.8%) reported a BRPE_peak_ value between 14/20 and 16/20, 6 participants (4.4%) a value between 11/20 and 13/20, 27 participants (20.1%) a value of 17/20, and 3 participants (2.2%) a value of 18/20. BRPE was not influenced by gender (*p* = 0.913 on the treadmill, *p* = 0.790 in the 1 km CTT).

### 3.2. BRPE in Relation to HR and V̇O_2_ during the Treadmill Walking Test

**Figure 1:**
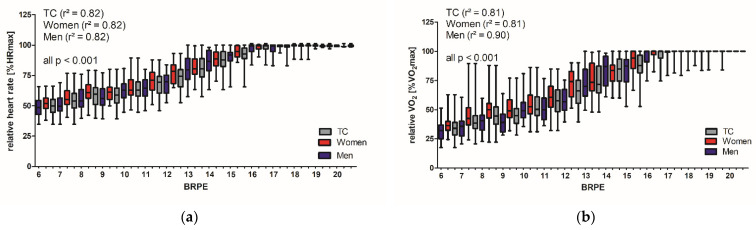
Measurements of exercise intensity and BRPE on the treadmill: differences between genders. (**a**) Relative HR (%HR_max_) and (**b**) relative V̇O_2_ (%VO_2max_) at different BRPE values for the total cohort (TC), women and men.

During the treadmill walking test, BRPE significantly correlated with relative HR (%HR_max_) (TC ρ = 0.88, men ρ = 0.88, women ρ = 0.89, all *p* < 0.001), as well as BRPE and relative V̇O_2_ (%V̇O_2max_) (TC ρ = 0.89, men ρ = 0.90, women ρ = 0.89 *p* < 0.001) at any given step (Figure 1).

For the estimation of physiological measures of exercise intensity, the cohort was divided into the sexes. Estimates for relative HR at each BRPE can be calculated according to the equation HR_men_ [%] = 20.85 + 4.35 x BRPE (adjusted r^2^ = 0.82, *p* < 0.001) and HR_women_ [%] = 27.11 + 4.13 × BRPE (adjusted r^2^ = 0.82, *p* < 0.001). Relative V̇O_2_ at each BRPE can be estimated according to the equation V̇O_2men_ [%] = −6.82 + 5.87 x BRPE (adjusted r^2^ = 0.90, *p* < 0.001) and V̇O_2women_ [%] = 4.78 + 5.40 × BRPE (adjusted r^2^ = 0.81, *p* < 0.001). Significant differences between the regression equations for men and women were found (*p* < 0.001).

### 3.3. BRPE in Relation to HR and V̇O_2_ during the 1 km CTT

**Figure 2:**
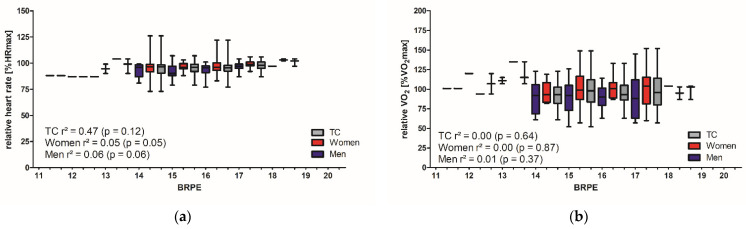
Measurements of exercise intensity and BRPE during the 1 km CTT: differences between genders. (**a**) Relative HR (%HR_max_) and (**b**) relative V̇O_2_ (%VO_2max_) at different maximal BRPE values for the total cohort (TC), women and men.

During the 1 km CTT, only a small correlation was observed between BRPE_max_ and %HR_max_ (TC ρ = 0.24, *p* < 0.01; men ρ = 0.26, *p* < 0.05; women ρ = 0.26, *p* < 0.05), respectively %V̇O_2max_ (TC ρ = 0.24, *p* < 0.01; men ρ = 0.30, *p* < 0.05). No significant correlation was found for the %V̇O_2max_ for women (Figure 2).

## 4. Discussion

This is the first study investigating the relationship between BRPE and objective measures of exercise intensity during uphill walking on the treadmill and in a natural environment.

The participants represent a healthy and fit study group. Male participants showed a mean relative V̇O_2max_ of 42.8 ± 8.6 mL·min^−1^·kg^−1^ during the treadmill walking testing and according to the American College of Sports Medicine’s (ASCM) guidelines for exercise testing and prescription corresponds with an excellent fitness level [56]. Female participants showed a mean relative V̇O_2max_ of 36.6 ± 5.8 mL·min^−1^·kg^−1^ and according to the ASCM guidelines corresponds with a superior fitness level. The present work demonstrated a high validity of the study group in assessing their perceived exertion during a graded walking test in the laboratory. During exercise interventions, it is important that individuals are capable of complying with the given exercise prescriptions and therefore of adhering independently to exercise intensities [43,57,58,59]. However, BRPE assessment was less valid during uphill walking in a natural environment. Even if the influence of exercise experience should not be underestimated, several studies [60,61,62] showed that trained and untrained people assess their intensity during training comparably well, regardless of age. Therefore, our findings with fit and healthy adults can also be assigned to healthy adults with a lower fitness level than the participants in our study.

Since HR is a valid and easy way to measure relative exercise intensity, many researchers examined the correlations between BRPE and HR to assess the criterion validity of BRPE. So far, most of the studies have investigated the relationships between subjective and objective measures of exercise intensity during cycling or running indoors. They showed high correlations between BRPE and HR, blood lactate, and/or V̇O_2_ [21,22,23,25]. Only a few studies have investigated the comparison of subjectively assessed BRPE values and objective measures during walking indoors [63,64,65] showing lower correlations between HR and BRPE than our results. None of the studies examined the correlations during uphill walking or hiking in nature. Investigations were only rarely carried out both in the laboratory and in the field [66,67,68]. No study used uphill walking as an exercise mode in their research. Ceci and Hassmén [67] investigated the comparison of running on a treadmill and an outdoor track with physically active men. They used a BRPE production protocol, where participants were asked to regulate their exertion related to BRPE of 11, 13, and 15. The study also showed lower correlations between HR and BRPE on the outdoor track compared to their treadmill running test. Furthermore, they suspected the influence of the environment on BRPE levels.

On a bicycle ergometer, Borg [17] found among the significant correlations the highest correlation value between BRPE and HR among teenagers (*r* = 0.70 to 0.90) and the lowest correlation value among 50 to 68 years old active men (*r* = 0.60). However, the present work showed a stronger correlation for HR and BRPE with *r* = 0.88 (*p* < 0.001) in elderly adults [median age of 56 years (IQR 52–63)] during uphill walking on a treadmill. With a comparable age group, the study of Miller, Bell, Collis and Hoshizaki [63] examined 202 post-50-year-old adults in a timed 600 m walk and a 2 min on-the-spot walk. They showed lower correlations between BRPE and HR (*r* = 0.25 to *r* = 048, *p* < 0.05). A meta-analysis by Chen, Fan, and Moe [23] demonstrated a mean validity coefficient of 0.62 for HR and BRPE (*n* = 3708) and 0.67 for %V̇O_2max_ and BRPE (*n* = 549). The very high correlation between BRPE and relative HR on the treadmill walking test in our study indicates a high validity of the predictive value of the objective measures of exercise intensity (HR and V̇O_2_) as a function of BRPE during the treadmill walking test (Figure 1). Our regression analysis explained 81% of the variance in %HR_max_ and %V̇O_2max_ with BRPE during the treadmill walking test. Compared to our results, Scherr, Wolfarth, Christle, Pressler, Wagenpfeil, and Halle [21] explained 55% of the variance in HR with BRPE. In our study, BRPE was not influenced by gender, which supports previous findings [21,69,70].

Several studies have already investigated and demonstrated the influence of nature on subjective perception. Krinski, Machado, Lirani, DaSilva, Costa, Hardcastle, and Elsangedy [68] reported lower ratings of perceived exertion during outdoor exercise in women with obesity. The environmental setting when walking outdoors has influenced BRPE. Participants of our study have shown nearly the same maximal respiratory exchange ratio (RER_max_) during the treadmill walking test and the 1 km CTT (Table 3), while they rated their subjectively perceived exertion lower in the 1 km CTT. This result indicates an existing influence of the natural environment on BRPE. In the literature, it is indicated that perceived pleasure while exercising with self-selected intensity plays an important role in being physically active [71,72,73]. Additionally, it has been repeatedly reported that people tend to walk faster outside than on the treadmill [74,75,76,77], which we have also confirmed with our results. Dasilva, Guidetti, Buzzachera, Elsangedy, Krinski, De Campos, Goss, and Baldari [77] approved the influence of the environment on walking at a self-selected pace. Given that when exercising outdoors, people tend to underestimate their exercise intensity, not so much in healthy subjects, but certainly, in patients, this has to be accounted for when prescribing exercise training outdoors. For preventive measures outdoors, such as the 1 km CTT, which requires a self-selected speed, we recommend a lower specification of the subjective assessment of exercise intensity be made to represent an essential safety measure. Moreover, adding sensor-based, objective exercise-intensity parameters such as HR holds promise to improve intensity prescription and health safety during uphill walking in a natural environment.

In summary, BRPE is a valid tool for healthy adults to monitor and prescribe exercise intensity in a laboratory setting. It should be determined whether our findings can be observed in a patient population. Our results support the use of BRPE in laboratory exercise tests with a healthy population. The influence of a natural environment on BRPE needs to be considered in future exercise interventions. For accurate intensity guidance, BRPE should be used in addition to HR monitoring in public health practice. Furthermore, future research is recommended for a detailed description of the relationship between subjective and objective measures of exercise intensity in an outdoor setting, also with regard to high altitudes (>3000 m).

### 4.1. Study Limitations

The current study has certain limitations. First, the participants of the study were all healthy without any known chronic disease. Additionally, they had a very good endurance capacity. Transferring our results from healthy adults to people with CDV risk factors or chronic diseases must be done with caution. Moreover, we identified large SD for HR and V̇O_2_ (Figure 1 and Figure 2), but according to further investigations, the standard error of the mean was quite small, demonstrating high accuracy of the estimated measures.

### 4.2. Perspective

The present work supports the BRPE scale as a useful tool for healthy adults to estimate and monitor their exercise intensity in the laboratory during a standardized graded walking test on the treadmill. Therefore, BRPE is a valid tool for prescribing exercise intensities during treadmill uphill walking indoors such as used in gyms or rehabilitation centers. However, during outside uphill walking lower Borg values should be recommended to achieve the right exercise intensity, especially for inactive or inexperienced people.

## 5. Conclusions

This is the first study investigating the relationships between the BRPE 6–20 scale and exercise intensity–assessed by heart rate and oxygen consumption–both during treadmill testing as well as during outdoor uphill walking. The present work demonstrated the value of using BRPE scales for the prescription of exercise intensity during uphill walking through subjective control of physical performance among healthy adults. This work identified a very high correlation between subjective and objective parameters of exercise intensity in the laboratory, where standardized protocols are used. A natural environment reduced criterion validity of BRPE during uphill walking and needs to be further investigated, especially in CVD patients with increased risk for adverse events.

## Figures and Tables

**Table 1:** The modified Bruce protocol (modification of the original protocol of Bruce [46]).

	Percent Grad (%)	Speed (km·h^−1^)
Stage 1	0	2.7
Stage 2	5	2.7
Stage 3	10	2.7
Stage 4	12	4.0
Stage 5	14	5.4
Stage 6	16	6.7
Stage 7	18	8.0
Stage 8	20	8.8

**Table 2:** Borg’s 15-grade scale for ratings of perceived exertion with values ranging from 6 to 20 (modified table according to Borg [52]).

6	
7	Very, very light
8	
9	Very light
10	
11	Fairly light
12	
13	Somewhat hard
14	
15	Hard
16	
17	Very hard
18	
19	Very, very hard
20	

**Table 3:** Characteristics of the study population.

	Total Cohort (*n* = 134)	Men (*n* = 58)	Women (*n* = 76)	*p*
**Anthropometrics** Age (years)	56 [52–63]	56 [52–62]	57 [51–63]	<0.731 ^1^
Height (cm)	171.6 [8.4]	178.4 [6.9]	166.5 [5.2]	
Weight (kg)	71.1 [13.3]	80.6 [11.2]	63.8 [9.8]	
BMI (kg/m^2^)	24.0 [3.4]	25.3 [2.8]	23.0 [3.5]	<0.001 *^,1^

Note: Data are shown as mean (SD) for normally distributed data or median (IQR 25th–75th percentile) for non-normally distributed data. * *p* < 0.05; ^1^ Student’s *t*-test Abbreviations: BMI, body mass index.

**Table 4:** Performance parameters during the maximal laboratory testing on the treadmill and the submaximal 1 km field testing.

	Total Cohort (*n* = 134)	Men (*n* = 58)	Women (*n* = 76)	*p*
**Exercise capacity on the treadmill**				
V̇O_2max_ (mL·min^−1^·kg^−1^)	39.3 [7.7]	42.8 [8.6]	36.6 [5.8]	<0.001 *^,1^
HR_max_ (bpm)	165 [14]	165 [16]	165 [12]	0.834 ^1^
RER_max_	1.1 [0.1]	1.1 [0.1]	1.1 [0.1]	0.907 ^1^
Borg_max_	18 [17–19]	18 [17–19]	17 [17,18]	0.180 ^2^
v_max_ (km·h^−1^)	5.6 [5.0–5.9]	5.8 [5.6–6.3]	5.4 [4.9–5.8]	<0.001 *^,2^
**Exercise capacity in the 1 km CTT**				
V̇O_2peak_ (mL·min^−1^·kg^−1^)	37.3 [6.3]	40.2 [6.1]	35.1 [5.6]	<0.001 *^,1^
HR_peak_ (bpm)	157 [15]	154 [15]	160 [14]	<0.01 *^,1^
RER_peak_	1.1 [1.0–1.2]	1.1 [1.0–1.2]	1.1 [1.0–1.1]	0.724 ^2^
Borg_peak_	15 [15,16]	16 [15,16]	15 [15,16]	0.687 ^2^
v_peak_ (km·h^−1^)	6.4 [5.7–7.2]	6.8 [5.9–7.3]	6.3 [5.4–7.0]	<0.05 *^,2^

Note: Data are shown as mean (SD) for normally distributed data or median (IQR 25th–75th percentile) for non-normally distributed data. * *p* < 0.05; ^1^ Student’s *t*-test; ^2^ Mann-Whitney U test. Abbreviations: V̇O_2max_, maximal oxygen uptake; HR_max_, maximal heart rate; RER_max_, maximal respiratory exchange ratio; Borg_max_, maximal Borg value; v_max_, maximal speed; V̇O_2peak_, peak oxygen uptake; HR_peak_, peak heart rate; RER_peak_, peak respiratory exchange ratio; Borg_peak_, peak Borg value; v_peak_, peak speed.

## Data Availability

The data presented in this study are available on request from the corresponding author.

## Abbreviations

%HR_max_	Relative maximal heart rate
%V̇O_2max_	Relative maximal oxygen consumption
CPET	Cardiopulmonary exercise testing
CRF	Cardiorespiratory fitness
CTT	Cardio-trekking test
CVD	Cardiovascular disease
HR	Heart rate
HR_max_	Maximal heart rate
PA	Physical activity
RER	Respiratory exchange ratio
RER_max_	Maximal respiratory exchange ratio
BRPE	Borg’s rating of perceived exertion
BRPE_max_	Maximal value of Borg’s rating of perceived exertion
TC	Total cohort
V̇O_2_	Oxygen consumption
V̇O_2max_	Maximal oxygen consumption (treadmill)
V̇O_2peak_	Peak oxygen consumption (1 km CTT)
